# Ginsenoside Derivative AD-1 Suppresses Pathogenic Phenotypes of Rheumatoid Arthritis Fibroblast-like Synoviocytes by Modulating the PI3K/Akt Signaling Pathway

**DOI:** 10.3390/cells14201625

**Published:** 2025-10-18

**Authors:** Yuan Fu, Fangfang Li, Biao Cui, Zhongyu Zhou, Xizhu Fang, Shengnan Huang, Xingguo Quan, Yuqing Zhao, Dan Jin

**Affiliations:** 1 Immunology Biology Key Laboratory, Yanbian University, Yanji 133002, China; yfu1990@126.com (Y.F.); ffli@ybu.edu.cn (F.L.); 15036815644@163.com (B.C.); 0000007941@ybu.edu.cn (X.F.); 15053791276@163.com (S.H.); quanxg121@ybu.edu.cn (X.Q.); 2 Key Laboratory of Natural Medicines of the Changbai Mountain, Ministry of Education, Yanbian University, Yanji 133002, China; zyzhou21@126.com; 3 Department of Immunology and Pathogenic Biology, College of Medicine, Yanbian University, Yanji 133002, China

**Keywords:** ginsenoside derivative AD-1, rheumatoid arthritis, network pharmacology, molecular docking, molecular dynamics, PI3K/Akt signaling pathway

## Abstract

**Highlights:**

- Network pharmacology identified that the key pathway for AD-1 in treating RA is PI3K/AKT.
- AD-1 inhibits the proliferation, migration, and invasion and promotes apoptosis of MH7A cells through the PI3K/AKT signaling pathway.
- AD-1 is a potential drug for treating RA, providing experimental evidence for the application of traditional Chinese medicine in the clinical treatment of RA.

**Abstract:**

Rheumatoid arthritis (RA) is a systemic autoimmune disorder marked by chronic inflammation of small synovial joints, with frequent extra-articular involvement of the skin and eyes. Prolonged methotrexate therapy for RA is often accompanied by serious side effects. Therefore, new drugs with less toxicity and greater effectiveness need to be developed. The ginsenoside 20(R)-25-methoxyl-dammarane-3β,12β,20-triol (AD-1), purified from *Panax ginseng* berry, exhibits potent anti-inflammatory and anti-cancer activities. However, the pharmacological mechanism of AD-1 in RA remains unclear. This study explored the potential anti-RA effects of AD-1 using an integrative strategy that combined network pharmacology, molecular docking, molecular dynamics simulation, and in vitro pharmacological validation. Enrichment analyses of KEGG and GO terms based on network pharmacology pointed to the PI3K/Akt signaling axis as a key regulatory pathway modulated by AD-1. Molecular docking and dynamics simulations revealed that AD-1 may have a close interaction with PIK3R1 and AKT1, demonstrating a stabilizing effect. Then, after experimental verification using human rheumatoid arthritis fibroblasts (MH7A), it was found that AD-1 suppressed cell proliferation, migration, and invasion and promoted apoptosis. Subsequent analysis of the RABC databases revealed that PIK3R1 and AKT1 were upregulated in RA, while AD-1 reduces phosphorylation of PI3K and Akt. In conclusion, these findings indicate that AD-1 exerts its anti-RA action, at least in part, through modulation of the PI3K/Akt signaling pathway and induction of apoptosis in synovial cells. This study provides a basis and new strategies for the role of ginsenosides in the treatment of RA.

## 1. Introduction

Rheumatoid arthritis (RA) is a chronic, systemic autoimmune disorder primarily targeting joints and periarticular soft tissues [1]. Clinically, RA is characterized by pain, swelling, morning stiffness, and progressive joint dysfunction. RA affects between 0.3% to 1% of the global population [2,3]. Although current treatment regimens include nonsteroidal anti-inflammatory drugs, disease-modifying anti-rheumatic drugs, and glucocorticoids, often accompanied by severe side effects [4,5]. Cyanidin occurs mainly as anthocyanins (O-glycosides), and that species and tissue-specific glycosylation, acylation and O-methylation produce multiple cyanidin derivatives by varying sugar identity and linkage position (3-O, 5-O or 7-O) and acylation patterns [6]. Recent studies have shown that many traditional Chinese medicines (TCMs) contain active ingredients such as Cyanidin, which have certain therapeutic effects. Such as Huangqi (*Astragalus membranaceus*), Guizhi (Twig of *Cinnamomum cassia*), Shaoyao (*Paeonia lactiflora*), and Zhimu (*Anemarrhena asphodeloides*) ameliorate rheumatoid arthritis pathology in preclinical models, as evidenced by reductions in inflammation and joint damage with Gui-Zhi-Shao-Yao-Zhi-Mu decoction and Huangqi Guizhi Wuwu decoction [7,8,9,10]. Nonetheless, given that many TCM have not yet been fully developed into standardized, clinically approved therapies, there remains an urgent demand for novel therapeutic agents with reliable efficacy, minimal adverse effects, and favorable tolerability profiles [11].

Ginsenosides, the primary active constituents of ginseng, have been demonstrated to exhibit diverse pharmacological properties, including anti-tumor and anti-inflammatory effects [12,13]. It has been documented that certain ginsenosides such as compound K and Rg3 have exhibited anti-RA potential [14,15]. However, given the structural diversity and pharmacological heterogeneity of ginsenosides, many derivatives remain unexplored, and identifying novel derivatives may uncover additional therapeutic opportunities or mechanisms. 20(R)-25-methoxyldammarane-3β, 12β, 20-triol (AD-1) is a dammarane-type triterpene sapogenin extracted from the hydrolyzed saponins of *Panax ginseng* berry [16,17,18,19]. AD-1 has shown promising biological, such as anti-pulmonary fibrosis, and anti-cancer effects [20,21,22]. Our previous work revealed that AD-1 has a favorable anti-inflammatory bowel disease effect. However, the anti-RA effect of AD-1 remains to be elucidated. Therefore, further studies are required to explore the roles and underlying mechanisms of AD-1 in RA.

In this study, we employed an integrated approach combining network pharmacology, molecular docking and molecular dynamics simulations to predict potential targets of AD-1 for the treatment of RA. The molecular mechanisms of these effects were then validated in vitro using MH7A cells by CCK-8, EdU incorporation, colony formation, scratch wound, transwell, tunnel, and Western blot (WB) assays. It is hoped that these research results can make the treatment of RA with ginsenosides possible.

## 2. Materials and Methods

### 2.1. Reagents

Recombinant human TNF-α (Peprotech 300-01A, Cranbury, NJ, USA), LY294002 (HY-10108), and 740Y-P (HY-P0175) were obtained from MCE (Shanghai, China). Dulbecco’s Modified Eagle’s Medium (DMEM), fetal bovine serum (FBS), penicillin, and streptomycin were purchased from Biological Industries (Beit HaEmek, Israel). CCK-8, EdU kit, and Tunel kit were provided by Beyotime (Shanghai, China). ECL detection reagent was purchased from Everbright (San Ramon, CA, USA). Primary antibodies against Bax (ab32124) and Bcl-2 (ab32124) were obtained from Abcam (Shanghai, China), and p-PI3K (17366), PI3K (4257), p-Akt (4691), Akt (4060), and β-actin (8457) were obtained from Cell Signaling Technology (Boston, MA, USA). HRP-conjugated goat anti-rabbit and anti-mouse IgG secondary antibodies were purchased from ZSGB-BIO (Beijing, China).

### 2.2. Potential Targets of AD-1

Potential molecular targets of AD-1 were predicted using PharmMapper server (https://www.lilab-ecust.cn/pharmmapper/index.html) (accessed on 14 March 2024) [23]. Corresponding UniProt ID and gene names were retrieved from the UniProt database (https://www.uniprot.org/) (accessed on 14 March 2024).

### 2.3. Identification of RA-Related Gene Targets

The search terms “rheumatoid arthritis” was entered into Online Mendelian Inheritance in Man (OMIM, https://omim.org/) database [24], GeneCards (https://www.genecards.org/) (accessed on 14 March 2024) database [25], and Therapeutic Target Database (http://db.idrblab.net/ttd/) (accessed on 14 March 2024) to retrieve the RA-related targets. Results obtained from the selected databases were merged, and redundant entries were excluded to generate a refined set of candidate targets associated with RA.

### 2.4. Target Prediction of AD-1 Against RA

The predicted targets of AD-1 and RA-related genes were uploaded to the VENNY2.1 website (https://bioinfogp.cnb.csic.es/tools/venny/index.html) (accessed on 14 March 2024) to identify overlapping genes These intersecting genes were regarded as potential therapeutic targets of AD-1 for the treatment of RA.

### 2.5. Network Analysis of Protein–Protein Interaction (PPI)

The overlapping targets were imported into the STRING database (https://string-db.org/) [26] to construct a PPI network. Network visualization and analysis were conducted using Cytoscape software (version 3.8.2) combined with CytoHubba plug-ins. Nodes were ranked using the degree algorithm, and the top 10 core genes were selected for further analysis. Functional enrichment analysis of AD-1–associated genes. To further characterize the biological functions of the predicted targets of AD-1, Gene Ontology (GO) enrichment analysis was performed the database for annotation, visualization, and integrated discovery (DAVID) (https://davidbioinformatics.nih.gov/home.jsp/) (accessed on 14 March 2024) [27]. Enriched terms were classified into three Gene Ontology categories: biological process (BP), cellular component (CC), and molecular function (MF), to clarify the functional roles of the predicted targets. In addition, Kyoto Encyclopedia of Genes and Genomes (KEGG) pathway analysis was performed using the KOBAS 3.0 tool (https://bioinfo.org/kobas) (accessed on 14 March 2024) [28] to identify key signaling pathways potentially modulated by AD-1. Subsequently, the resulting enrichment data were subsequently imported into the online platform (http://www.bioinformatics.com.cn/) (accessed on 14 March 2024) for graphical visualization and functional annotation.

### 2.6. Molecular Docking Verification

Obtain the 2D structure of the small molecule ligand (AD-1: 20(R)-25-Methoxyl-Dammarane-3Beta,12Beta,20-Triol, PubChem CID 53322140, Molecular Formula C_30_H_54_O_4_) from the PubChem database (http://pubchem.ncbi.nlm.nih.gov/), input the 2D structure into Chem Office 20.0 software to create its 3D structure and save it as a mol2 file. Then, use the RCSB PDB database (http://www.rcsb.org/) to screen protein targets with higher resolution crystal structures as molecular pairs for the receptor (PIK3R1 (PDB: 4WAF Resolution: 2.39 Å), AKT1 (PDB: 3QKM Resolution: 2.2 Å)). Use PyMOL 2.6.0 software to perform operations such as dehydration and dephosphorylation on the protein, and save it as a PDB file. Use AutoDock software 1.1.2 to minimize the energy of the compound and preprocess the target protein to search for the active pocket. Finally, run AutoDock for molecular docking, with the number of iterations set to 50. Based on the binding energy size, evaluate the binding activity of the two, and visualize the results using PyMOL 2.6.0 and Discovery Studio 2019 software.

### 2.7. Molecular Dynamics Simulation

Based on the small molecules and protein complexes (AD-1-PIK3R1, AD-1-AKT1) obtained through the above docking, full-atom molecular dynamics simulations were conducted for each of them using the AMBER 18 software (An overview of the Amber biomolecular simulation package). Before the simulation, the charges of the small molecules were calculated using the antechamber module and Gaussian 09 software’s Hartree-Fock (HF) SCF/6-31G* method. Subsequently, the small molecules and proteins were described using the GAFF2 small molecule force field and the ff14SB protein force field (ff14SB: improving the accuracy of protein side chain and backbone parameters from ff99SB, development and testing of a general amber force field). Each system was given hydrogen atoms using the LEaP module, and a truncated octahedral TIP3P solvent box was added at a distance of 1 nm in the system (Structure and dynamics of the TIP3P, SPC, and SPC/E water models at 298 K). Na^+^/Cl^−^ was added to the system to balance the charge, and the topology and parameter files for the simulation were output.

Before the simulation, the energy of the system was optimized, including 2500 steps of steepest descent method and 2500 steps of conjugate gradient method. After the energy optimization of the system was completed, the system was heated at a fixed volume and a constant heating rate for 200 ps to slowly raise the temperature from 0 K to 298.15 K. Under the condition of maintaining the temperature at 298.15 K, a 500 ps NVT (isothermal-isobaric) simulation was performed to further evenly distribute the solvent molecules in the solvent box. Subsequently, a 500 ps NPT (isothermal-isobaric) equilibrium simulation was conducted for the entire system. After that, the two complex systems were subjected to 200 ns NPT (isothermal-isobaric) simulations under periodic boundary conditions. During the simulation, the cutoff distance for non-bonded interactions was set to 1 nm [29], and the Particle Mesh Ewald (PME) method was used to calculate the long-range electrostatic interactions (Molecular dynamics simulations of biomolecules: Long-range electrostatic effects) [30], and the SHAKE method was used to limit the bond lengths of hydrogen atoms (A fast SHAKE: Algorithm for solving distance constraint equations for small molecules in molecular dynamics simulations) [31], Langevin algorithm for temperature control (Langevin stabilization of molecular dynamics simulations of polymers using quasi-symplectic algorithms), where the collision frequency γ is set to 2 ps^−1^. The system pressure was 1 atm, the integration step size was 2 fs, and the trajectory was saved every 10 ps for subsequent analysis. At last, the system was subjected to 200 ns of molecular dynamics simulations under the NPT ensemble. Trajectory data were recorded at intervals of 10 ps, and correlation analysis was performed on the trjconv module. The free energy of binding between AD-1 and PI3KR1/AKT1 was determined following the MM/GBSA method.

### 2.8. Chemicals

AD-1 (25-OCH_3_-PPD; 20(R)-25-methoxyl-dammarane-3β,12β,20-triol) was kindly provided by Prof. Yuqing Zhao (School of Pharmacy, Yanbian University). The sample was obtained as colorless crystals, and the provider reported a purity of >97% [32]. AD-1 is a known dammarane-type sapogenin; its molecular formula C_31_H_56_O_4_ and characteristic mass-spectrometric signals (ESI-MS *m*/*z* 493 [M + H]^+^, 515 [M + Na]^+^; HR-MS *m*/*z* 515.4064, calcd for C_31_H_56_O_4_Na 515.4076) as well as full ^1^H and ^13^C NMR assignments have been reported previously and the reported specific rotation ([α]ᴰ^20^ = +8.0, c 0.1, MeOH) [16,17,18,19,33]. AD-1 was dissolved in dimethyl sulfoxide (DMSO; Sigma-Aldrich, MO, USA), aliquoted, and stored at −20 °C until use.

### 2.9. Cell Culture

The human RA-FLS cell line MH7A (BNCC342313) was purchased from the Bena Culture Collection (BNCC, Beijing, China). Cells were authenticated by short tandem repeat profiling and confirmed to be free of mycoplasma contamination using the MycoAlert™ Mycoplasma Detection Kit (Lonza, Basel, Switzerland). MH7A cells were cultured in DMEM medium with 10% FBS at 37 °C in a humidified incubator containing 5% CO_2_. DMSO 0.625% in all assays.

### 2.10. Cell Viability Assay

MH7A cells were seeded into 96-well plates at a density of 5 × 10^3^ cells per well and cultured for 24 h. The cells were then treated with various concentrations of AD-1 for 24 or 48 h. After treatment, 10 μL of CCK-8 solution was added to each well and incubated for 2 h. The absorbance at 450 nm was measured using a microplate reader to evaluate cell viability.

### 2.11. 5-Ethynyl-2′-deoxyuridine (EdU) Incorporation Assay

MH7A cells were seeded in 96-well plates at 5 × 10^3^ cells per well and treated with TNF-α and AD-1 for 24 h. After treatment, the cells were fixed, washed, and incubated with EdU medium, followed by a click reaction according to the manufacturer’s instructions, and finally stained with Hoechst 33342 (Beyotime, Shanghai, China) for nuclear counterstaining. The proportion of EdU-positive cells was determined using fluorescence microscopy (Olympus, Tokyo, Japan) and calculated as the percentage of EdU-positive cells relative to the total number of Hoechst 33342-stained nuclei.

### 2.12. Colony Formation Assay

MH7A cells were seeded into 6-well plates at a density of 500 cells per well and treated with TNF-α and AD-1 for 24 h. After treatment, the medium was replaced with fresh DMEM without TNF-α or AD-1, and the cells were cultured for an additional 7 days. At the end of the incubation period, the cells were fixed and stained with Giemsa solution for 15 min. Colonies were photographed and manually counted under a light microscope.

### 2.13. Migration and Invasion Assay

The cells were implanted into a 6-well plate of 2 × 10^5^ cells/well. Monolayer cells were scraped with a sterile pipette to form the wound, washed with PBS 3 times. The cells were then incubated with TNF-α and/or AD-1 solution, and images of the wound area were captured at 0, 12, and 24 h to evaluate wound closure. The wound size and migration distance were calculated.

For the invasion assay, a cell suspension with a density of 5 × 10^3^ cells/100 μL was prepared using a culture medium containing 1% FBS, and it was inoculated into the upper chamber. In the lower chamber, 600 μL of culture medium containing 1% FBS was added to each well, and then the samples were placed in a cell culture box for cultivation. The next day, the culture medium in the lower chamber containing 1% FBS was replaced with one containing 10% FBS, and the upper chamber was treated with TNF-α and/or AD-1. After incubation for 24 h, the cells were fixed, stained, photographed, and counted under a microscope.

### 2.14. Apoptosis Assay

The kit employs the one-step TUNEL apoptosis assay kit (Beyotime, Shanghai, China). The cells were implanted into a 24-well plate of 2 × 10^4^ cells/well. Place the samples in the incubator overnight. Add the drugs the next day. After 24 h, wash with PBS once, fix with 4% paraformaldehyde for 30 min, then wash with PBS, add the solution containing 3% Triton X-100 for incubation at room temperature for 5 min, prepare the TUNEL detection solution, 50 μL per well. Microscope photography.

### 2.15. RABC Database Analysis

Data from the RABC database (www.onethird-lab.com/RABC/) (accessed on 22 September 2024); dataset GSE45291-RABC39) were analyzed to examine the differential expression of genes in the PI3K/Akt signaling pathway between RA samples and healthy controls.

### 2.16. Western Blot

MH7A cells were seeded at 2 × 10^6^ cells per well in 60 mm plates and overnight, followed by treatment with various concentrations of AD-1 for an additional 24 h. Wash the cells with pre-cooled PBS (phosphate-buffered saline), add RIPA lysis buffer containing 1% protease inhibitor and 1% phosphatase inhibitor, lyse for 30 min, and collect the cells with a cell scraper. Make sure all operations are carried out on ice. Centrifuge at 4 °C for 30 min, then remove the supernatant and separated on 6–10% sodium dodecyl sulfate polyacrylamide gels, and transferred to polyvinylidene difluoride membranes as described previously [34]. The membranes were incubated overnight at 4 °C with primary antibodies against Bax (Abcam ab32124), Bcl-2 (Abcam ab32124), p-PI3K (CST-17366), PI3K (CST-4257), p-Akt (CST-4060), Akt (CST-4691), and β-actin (CST-8457). After washing, the membranes were incubated with HRP-conjugated secondary antibodies (Aleuronic, Beijing, China) for 1 h at room temperature. Protein bands were detected using ECL chemiluminescence reagents (Everbright, San Ramon, CA, USA).

### 2.17. Statistical Analysis

Statistical analyses were performed using GraphPad Prism version 7.0 (GraphPad Software, Inc., San Diego, CA, USA). All experiments were conducted in triplicate. Data were presented as the mean ± standard deviation (SD). Comparisons between two groups were performed using Student’s *t*-test, while multiple group comparisons were conducted using one-way analysis of variance (ANOVA). A *p*-value < 0.05 was considered statistically significant.

## 3. Results

### 3.1. PPI Network Analysis and Screening of Potential Targets of AD-1 Against RA

**Figure 1:**
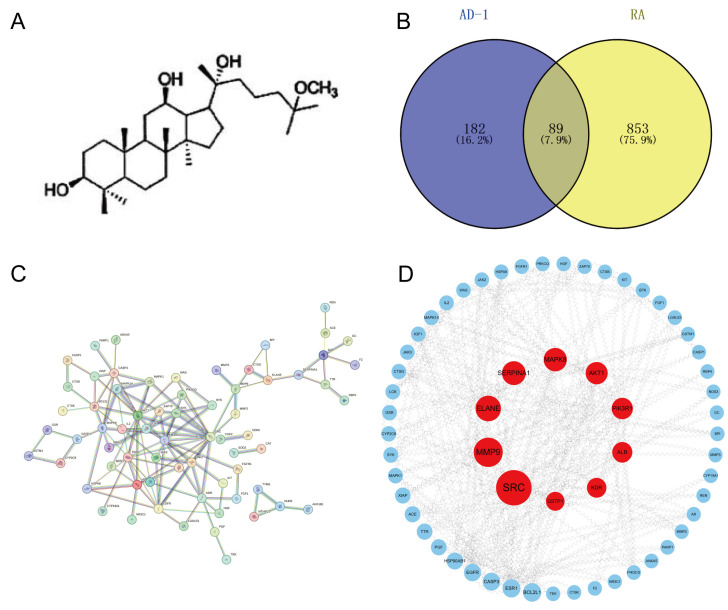
Identification of potential targets of AD-1 against RA and construction of the PPI network. (**A**) The chemical structure of AD-1. (**B**) Venn diagram illustrating 89 overlapping targets between AD-1–predicted targets and RA-related genes. (**C**) PPI network constructed from the 89 intersecting targets. (**D**) Top 10 hub genes identified and visualized in Cytoscape based on degree centrality; node color intensity (red) corresponds to higher connectivity.

The chemical structure of AD-1 is depicted in Figure 1A. To identify potential AD-1 targets relevant to RA, the structure of AD-1 was submitted to the PharmMapper databases, resulting in 271 potential targets. Concurrently, a total of 942 RA-related targets were retrieved from the OMIM, TTD, and GeneCards databases. The intersection between AD-1 targets and RA-related genes was determined using the VENNY2.1 tool, identifying 89 overlapping targets considered as potential candidates for AD-1 in the treatment of RA (Figure 1B, Table 1). To further investigate the interactions among these overlapping targets, the 89 genes were input into the STRING database to construct a PPI network, which comprised 89 nodes and 122 edges (Figure 1C). The PPI network subsequently visualized using the Cytoscape software 3.10.1 for visualization, and the top 10 hub genes were identified using the CytoHubba plugin based on the degree algorithm. As shown in Figure 1C and Table 2, the identified genes include SRC, MMP9, ELANE, SERPINA1, MAPK8, AKT1, PIK3R1, ALB, KDR, and GSTP1. Notably, PIK3R1 and AKT1 were among the top 10, indicating their potential to play a significant role in the anti-RA mechanism of AD-1.

### 3.2. GO and KEGG Pathway Enrichment Analysis

**Figure 2:**
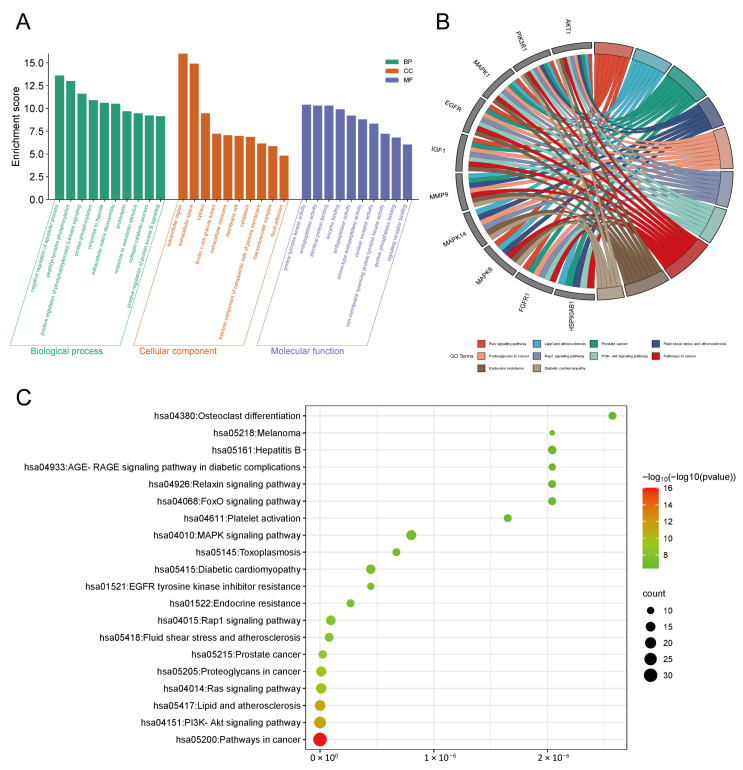
GO and KEGG pathway enrichment analyses of the intersecting genes. (**A**) GO enrichment analysis results, including BP, CC, and MF. (**B**) Chord diagram illustrating the association between core genes and enriched GO and KEGG pathways. (**C**) Bubble plot showing KEGG pathway enrichment results of the intersecting genes, analyzed using the KOBAS database.

To further elucidate the biological functions of the 89 overlapping targets of AD-1 against RA, GO enrichment analysis was performed using the DAVID database. The results revealed significantly enriched in 413 BPs, 50 CCs and 90 MFs (*p* < 0.05). As shown in Figure 2A, BP enrichment analysis indicated that AD-1 may be implicated in the regulation of the apoptotic process, peptidyl-tyrosine phosphorylation, phosphatidylinositol 3-kinase signaling, and protein phosphorylation. CC enrichment analysis revealed that AD-1 predominantly functions in the extracellular region, extracellular space, cytosol, and ficolin-1-rich granule lumen. Meanwhile, the MF enrichment analysis suggested that AD-1’s anti-RA effects might be involved in protein tyrosine kinase activity, endopeptidase activity, identical protein binding, and enzyme binding. Further analysis of the top 10 KEGG signaling pathways highlighted AKT1 and PIK3R1 as two of the most prominently involved proteins (Figure 2B). In addition, KEGG pathway enrichment analysis using the KOBAS database identified 130 significantly enriched pathways (*p* < 0.05), among which the PI3K/Akt signaling pathway and pathways in cancer were the most prominent (Figure 2C). Given the essential roles of PI3K/Akt in regulating cell proliferation, migration, and apoptosis, this pathway may represent a core mechanism through which AD-1 exerts therapeutic effects against RA.

### 3.3. Molecular Docking Simulation Techniques

**Figure 3:**
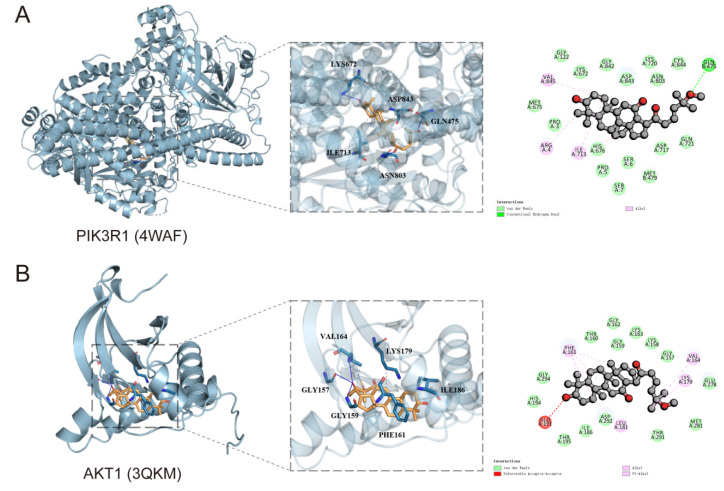
Molecular docking models of AD-1 with target proteins. (**A**,**B**) Predicted binding mode of AD-1 with PIK3R1 (4WAF) and AKT1 (3QKM), showing key residues involved in hydrogen bonding and hydrophobic interactions, along with the types of molecular interactions. The golden color represents the AD-1 chemical structure. Blue color represents the predicted binding sites.

Molecular docking simulation techniques have been demonstrated to predict the binding modes and binding affinities between small molecules and their target proteins, thereby assessing the potential interaction and selectivity of candidate compounds [35]. To investigate the binding interaction between AD-1 and key target proteins, docking analysis was conducted using AutoDock software. The parameters of the 4WAF grid box were (x: −0.308, y: 5.948, z: −16.621), and the parameters of the 3QKM were (x: 25.908, y: 0.839, z: 9.004). The results showed that AD-1 could bind to the potential binding sites of the target proteins, with docking energies were −8.1 kcal/mol (PIK3R1) and −8.4 kcal/mol (AKT1), as summarized in Table 3 and Table S1. In the literature it was reported that between inhibitors that are definitely active and inactive the threshold is −7.0 kcal/mol as reported by Chang et al. [36]. The docking results showed that both PIK3R1 and AKT1 have good binding activity. Thus, AD-1 displayed good binding affinity to both targets. As shown in Figure 3A, residues Gln475, Lys 672, Asn803, Ile713 and Asp843 of PIK3R1 bonds with AD-1. In AKT1, residues Gly157, Gly159, Phe161, Lys179, Val164, and Ile186 bonds with AD-1 (Figure 3B).

### 3.4. Molecular Dynamics Simulation

**Figure 4:**
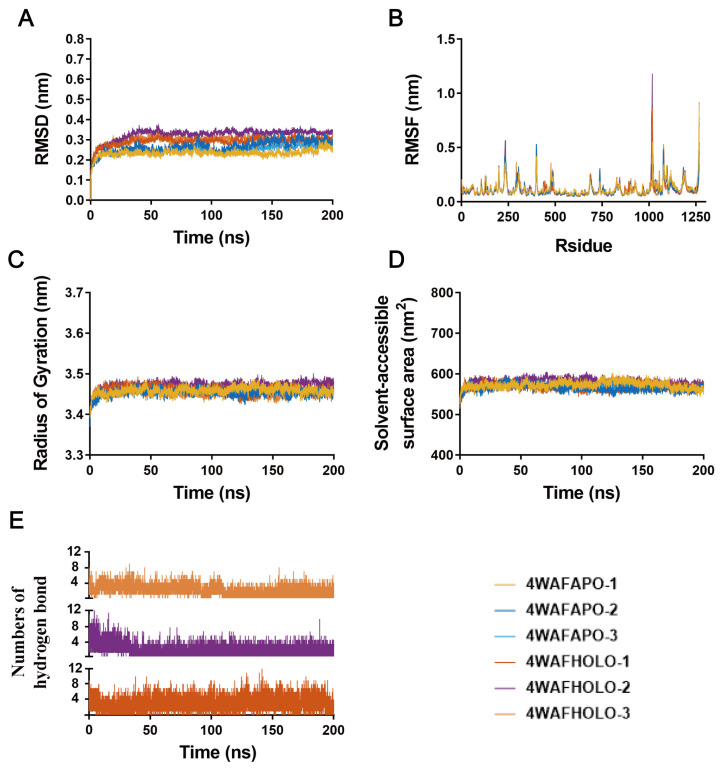
Time-dependent analysis of molecular dynamics trajectories of the PIK3R1–AD-1 complex. (**A**) Root-mean-square deviation (RMSD) of PIK3R1 (nm). (**B**) Root-mean-square fluctuation (RMSF) of PIK3R1 residues. (**C**) Radius of gyration (Rg) of PIK3R1 (nm). (**D**) Solvent-accessible surface area (SASA) of PIK3R1 (nm^2^). (**E**) Number of hydrogen bonds formed between AD-1 and PIK3R1 during the simulation. (*n* =3).

**Figure 5:**
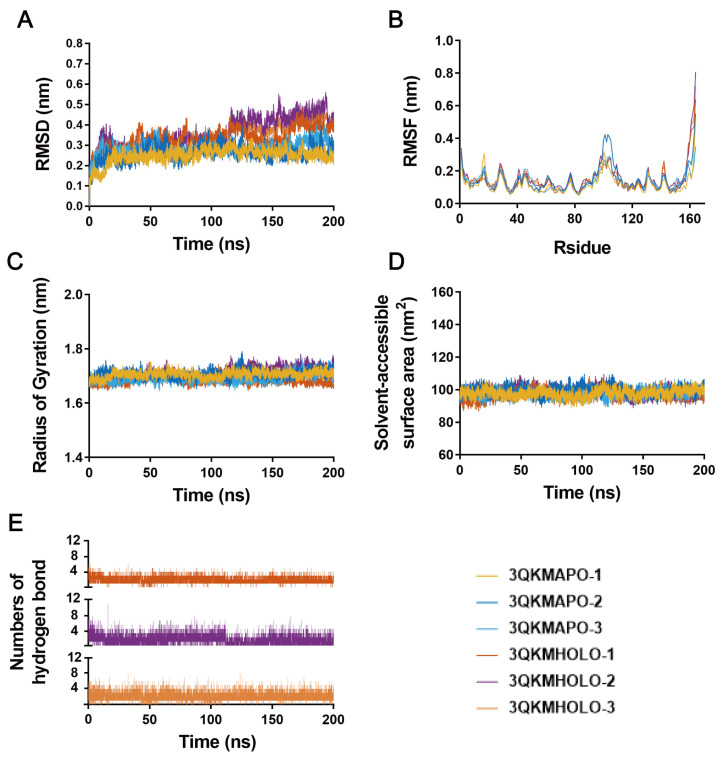
Time-dependent analysis of molecular dynamics trajectories of the AKT1–AD-1 complex. (**A**) RMSD of AKT1. (**B**) RMSF of AKT1 residues. (**C**) Rg of AKT1. (**D**) SASA of AKT1. (**E**) Number of hydrogen bonds formed between AD-1 and AKT1 during the simulation. (*n* = 3).

The results of molecular dynamics could more accurately assess the stability and conformational changes in protein–ligand binding, and could also capture details that cannot be predicted by experimental methods, thus providing a deeper exploration of the mechanism of action [37]. To explain the interaction between AD-1 and PIK3R1 (4WAF) and AKT1 (3QKM), we conducted a 200 ns molecular dynamics simulation. The root mean square deviation (RMSD) was used to characterize the conformational differences or trajectory stability of small molecules and proteins during the dynamic binding process. As shown in Figure 4A, the RMSD value of the PIK3R1 (4WAF)-AD-1 complex reached an equilibrium state of approximately 0.3 nm after 20 ns. As shown in Figure 5A, the RMSD of the AKT1 (3QKM)-AD-1 complex reached an equilibrium state of approximately 0.35 nm after 20 ns. It was worth noting that in the three results, AD-1 with PIK3R1 (4WAF) obtained results similar to 4WAF, but AKT1 (3QKM) showed a slight increase after 120 ns, but overall tended towards a stable equilibrium state. This indicates that AD-1 binds tightly to both PIK3R1 and AKT1 [38].

The root mean square fluctuation (RMSF) was used to describe the fluctuation of each atom and can be regarded as an indicator of the flexibility degree of the molecular structure [39]. Figure 4B showed that in the 4WAF-AD-1 complex, the fluctuation amplitude of the peptide residue was consistent with that of 4WAF. Figure 5B showed that in the 3QKM-AD-1 complex, the fluctuation amplitude of peptide residue 100 was significantly smaller than that of 3QKM. These results indicated that AD-1, when binding to both proteins, maintains a certain rigidity, without significant changes, and had good stability [40].

The radius of gyration (Rg) was used to measure the compactness of protein structure. A lower value indicated a more compact protein. According to the data in Figure 4C, within 200 ns, the Rg of 4WAF-AD-1 rapidly reached an equilibrium state and stabilized at 3.4–3.5 nm, while the Rg of 3QKM-AD-1 remained stable at around 1.7 nm (Figure 5C). This might be due to the formation of more effective interactions within the protein to better match AD-1, thereby promoting the stability of the complex.

The solvent accessible surface area (SASA) was used to characterize the folding stability of enzymes and the changes in their surface volume. The 4WAF-AD-1 complex (Figure 4D) and the 3QKM-AD-1 complex (Figure 5D) showed a stable trend, indicating that AD-1 maintains the size of the protein pocket and the surface area. Hydrogen bonds were used to characterize the interaction forces between small molecules and enzymes (mainly electrostatic forces in this system). During the entire dynamic process, the hydrogen bond interactions of the complexes alternate. The 4WAF–AD-1 complex formed approximately 4 to 6 hydrogen bonds on average (Figure 4E), whereas the 3QKM–AD-1 complex formed approximately 2 to 4 (Figure 5E). The number of hydrogen bonds in the three results of the two complexes was close, showing good repeatability, indicating the stability of the binding of AD-1 to the protein.

To investigate the primary driving forces in the dynamic process between AD-1 and PIK3R1 or AKT1, binding free energy was determined with MM/GBSA method [41]. As shown in Table 4, van der Waals interaction (ΔE_vdw_), the electrostatic interaction (ΔE_ele_) and non-polar solvation energy contributed positively to preserving the binding stability of AD-1. The three repeated MM/GBSA results indicated that AD-1 has shown an excellent binding affinity with PIK3R1 and AKT1, consistent with the molecular docking outcomes mentioned above.

### 3.5. AD-1 Inhibits Cell Proliferation, Migration and Invasion of MH7A Cells

**Figure 6:**
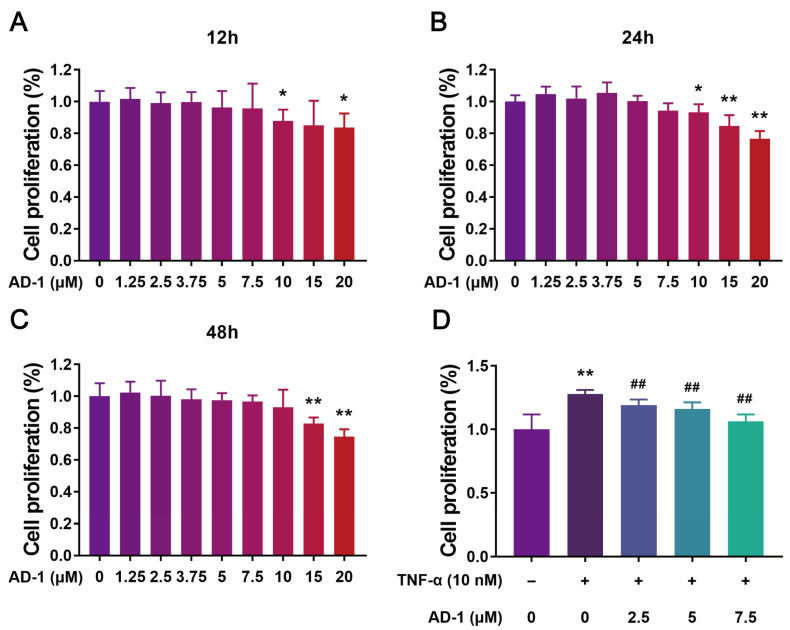
AD-1 inhibits the proliferation of MH7A cells. (**A**–**C**) Cell proliferation of MH7A cells was measured after treatment with various concentrations of AD-1 for 12 h, 24 h, and 48 h, respectively (*n* = 3). (**D**) Inhibitory effect of AD-1 on TNF-α-stimulated MH7A cell proliferation (*n* = 3). * *p* < 0.05, ** *p* < 0.01 vs. control group; ^##^ *p* < 0.01 vs. TNF-α group.

**Figure 7:**
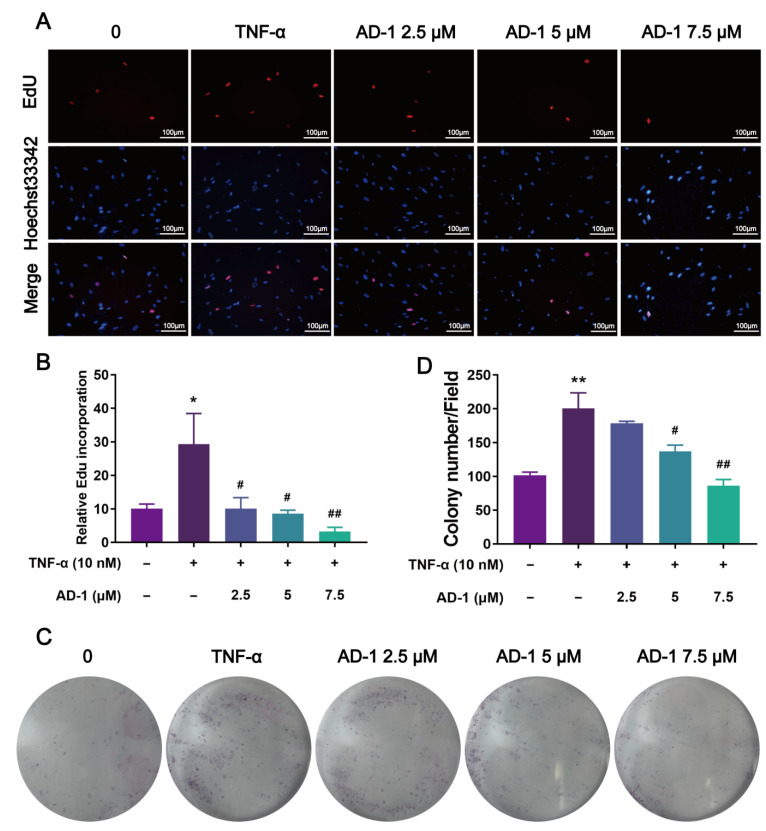
AD-1 inhibits the DNA replication and colony-forming ability of MH7A cells. (**A**,**B**) The DNA replication ability of MH7A cells was assessed by EdU assay (*n* = 3). (**C**,**D**) Colony formation assay was performed to assess the impact of AD-1 on the clonogenic capacity of MH7A cells (*n* = 3). * *p* < 0.05, ** *p* < 0.01 vs. control group; ^#^ *p* < 0.05, ^##^ *p* < 0.01 vs. TNF-α group.

**Figure 8:**
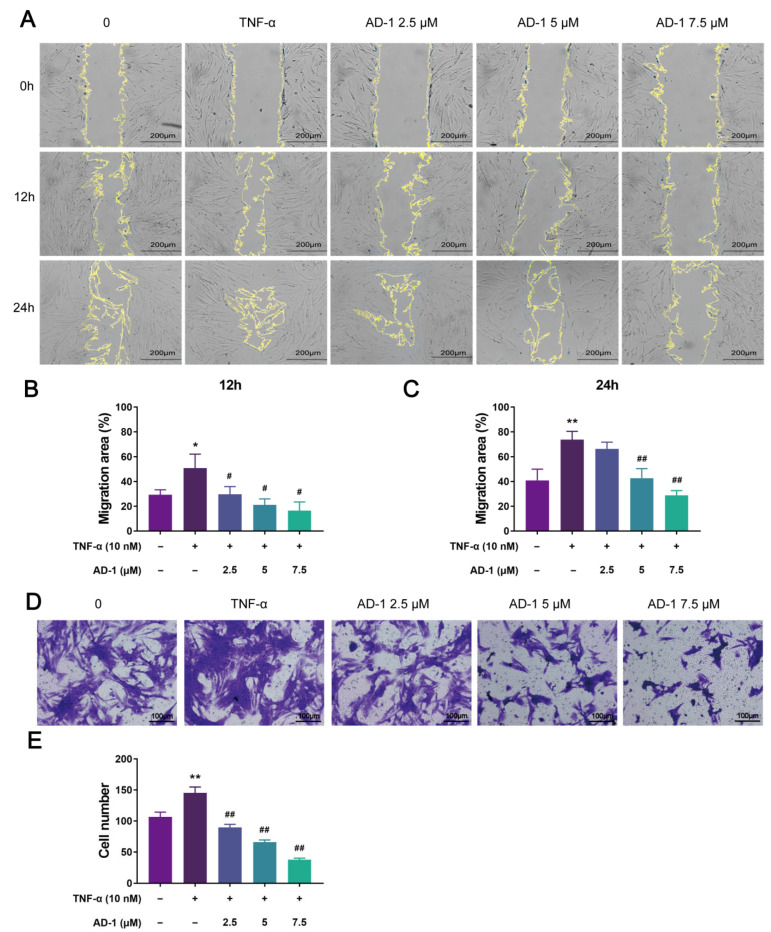
AD-1 inhibits MH7A cells migration and invasion. (**A**–**C**) Wound healing assay was performed to evaluate the effect of AD-1 on MH7A cell migration (*n* = 3). (**D**,**E**) Transwell assay was performed to evaluate the effect of AD-1 on MH7A cell invasion (*n* = 3). * *p* < 0.05, ** *p* < 0.01 vs. control group; ^#^ *p* < 0.05, ^##^ *p* < 0.01 vs. TNF-α group.

To verify the findings from network pharmacology, molecular docking and molecular dynamics, we performed a series of in vitro experiments using MH7A cells. Firstly, the CCK-8 assay revealed that AD-1 significantly inhibited TNF-α-stimulated proliferation of MH7A cells at non-cytotoxic concentrations (Figure 6A–D). To further investigate the impact of AD-1 on DNA replication, EdU assay was conducted, showing a dose-dependent inhibition of DNA synthesis (Figure 7A,B). Colony formation assays confirmed the anti-proliferative effect of AD-1, demonstrating a marked reduction in colony number (Figure 7C,D). In addition, the wound healing assay and transwell assay indicated that AD-1 significantly suppressed the migration and invasion ability of MH7A cells in a concentration-dependent manner (Figure 8).

### 3.6. AD-1 Induced Apoptosis of MH7A Cells

**Figure 9:**
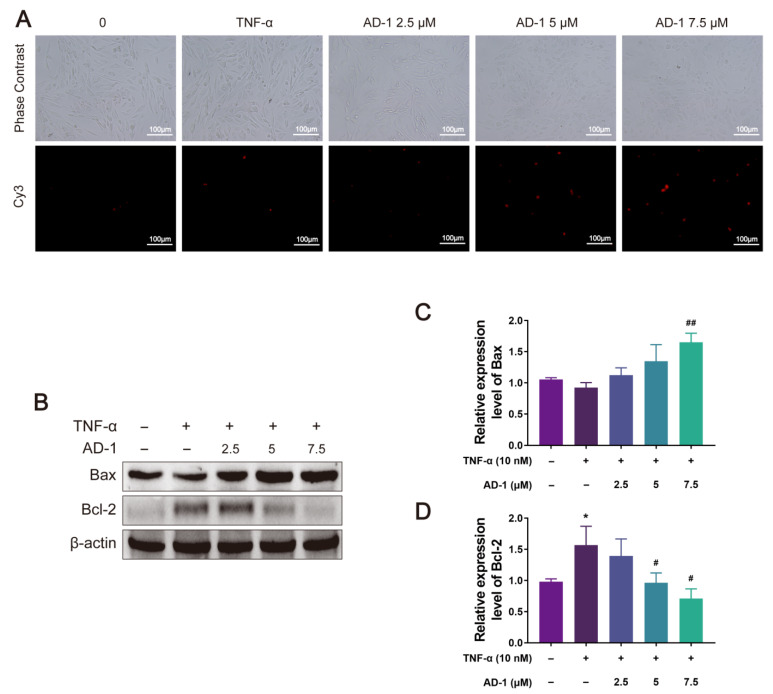
AD-1 induces MH7A cells apoptosis. (**A**) One-step TUNEL apoptosis assay was performed to evaluate the effect of AD-1 on MH7A cell apoptosis (*n* = 3). (**B**–**D**) The protein expressions of Bax, Bcl-2, and β-actin were detected by Western blot (*n* = 3). * *p* < 0.05 vs. control group; ^#^ *p* < 0.05, ^##^ *p* < 0.01 vs. TNF-α group.

The Bcl-2 protein family, which includes Bcl-2 and Bax, is a key regulatory protein family that initiates the process of apoptosis. In order to ascertain whether AD-1 induces apoptosis in MH7A cells, the expression levels of apoptosis-related proteins were assessed by Tunel assay and Western blot analysis. The results of the Tunel assay indicated that AD-1 could promote the apoptosis of MH7A cells (Figure 9A). Meanwhile, the Western blot results showed that AD-1 significantly downregulated the expression of Bcl-2 protein in a dose-dependent manner, while increasing the expression of Bax protein (Figure 9B–D).

### 3.7. AD-1 Inhibits the Activation of the PI3K/Akt Signaling Pathway in MH7A Cells

**Figure 10:**
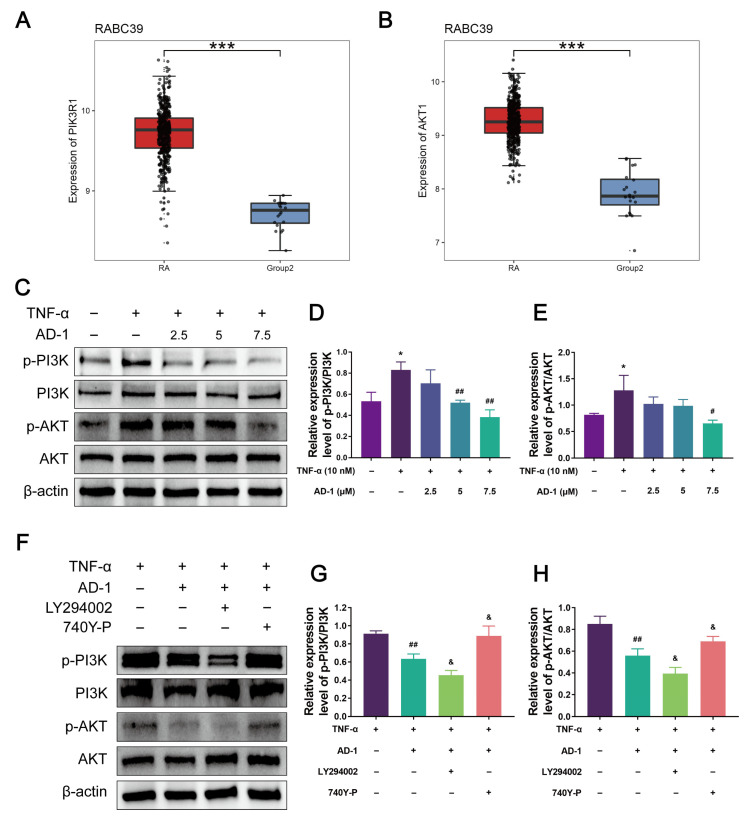
AD-1 inhibits the activation of PI3K/Akt signaling pathway. (**A**,**B**) The mRNA expression levels of PIK3R1 and AKT1 in RA and normal samples from the RABC database. RA samples are shown in red for *n* = 493; normal controls in blue for *n* = 20. A *t-test* was used for the statistics. *** *p* < 0.001. (**C**–**E**) Western blot analysis of phosphorylated and total forms of PI3K and Akt, indicating pathway activation status (*n* = 3). (**F**–**H**) The PI3K inhibitor LY294002 (20 μM) or the PI3K activator 740Y-P (5 μM), and changes in PI3K/Akt pathway activity were assessed (*n* = 3). * *p* < 0.05, vs. control group; ^#^ *p* < 0.05, ^##^ *p* < 0.01 vs. TNF-α group. ^&^ *p* < 0.05 vs. AD-1 group.

The above results demonstrated that AD-1 effectively inhibits cell proliferation and migration while promoting apoptosis. Based on network pharmacology analysis, the PI3K/Akt signaling pathway was predicted to be involved in the anti-RA effects of AD-1. To explore this hypothesis, the correlation between the PI3K/Akt signaling pathway and RA was analyzed using the RABC database. Notably, the mRNA expressions of PIK3R1 and AKT1 were significantly elevated in RA samples (n = 493) compared to healthy controls (n = 20) in Figure 10A,B, suggesting that PI3K and Akt are overexpressed in RA. To validate this finding, we evaluated the expression levels of PI3K, phosphorylated PI3K (p-PI3K), Akt, and phosphorylated Akt (p-Akt) in MH7A cells after AD-1 intervention. The results revealed that AD-1 markedly reduced the phosphorylation of both PI3K and Akt (Figure 9C), without significantly affecting total protein levels (Figure 10C–E). To validate these findings, we further employed the PI3K inhibitor LY294002 (20 μM) and the PI3K agonist 740Y-P (5 μM). Co-treatment with LY294002 further enhanced the inhibitory effects of AD-1 on PI3K and Akt phosphorylation whereas co-treatment with the PI3K activator 740Y-P partially restored their phosphorylation (Figure 10F–H). These findings indicate that the anti-RA effect of AD-1 is mediated through the PI3K/Akt signaling pathway.

## 4. Discussion

Rheumatoid arthritis (RA) is a prevalent autoimmune disease marked by chronic inflammation of the synovial membrane. Sustained inflammatory responses, if not adequately controlled, can accelerate cartilage and bone erosion, ultimately causing joint deformity and functional disability [42]. Methotrexate is a commonly used drug for the treatment of RA, but long-term use of methotrexate has serious side effects. So new drugs need to be developed that are less toxic and more effective. TCMs have a low toxicity and a multi-target mode of action. In recent years, increasing attention has been focused on the application of TCMs as a novel therapeutic approach for RA [43,44,45,46]. In TCMs, ginseng is one of the most representative Chinese herbs. Ginsenoside is the main component of ginseng, Wang et al. demonstrated that the ginsenoside compound K exerts joint-protective effects by activating GR and down-regulating the TNF-α-TNFR2 pathway, thereby influencing the proliferation, migration and secretion of FLS [47]. Yi’s review indicates that CK, G-Rb1, Pn-BE, and PNS ameliorate RA by decreasing MMP-1, MMP-9, MMP-13, TNF-α, IL-1β, iNOS, and NF-kB/MAPK signaling, clinical arthritis scores, cartilage destruction, immune cell infiltration, and onset/progression of RA and by improving arthritis clinical symptoms in various cells, animal models, and human RA patients [48]. AD-1 is a novel ginsenoside derivative with low toxicity and multi-target characteristics. Studies have shown that AD-1 has excellent drug metabolism performance [49], and also has anti-fibrotic [20], and anti-tumor properties [19], including anti-lung cancer [22], anti-gastric cancer [50], anti-colon cancer [21], anti-ovarian cancer [33,49], etc. Previous studies have demonstrated that AD-1, as well as other ginsenoside derivatives, can inhibit the PI3K/Akt pathway, thereby suppressing abnormal cell proliferation and promoting apoptosis in various disease models including cancer. This indicates that PI3K/Akt may be a common and crucial signaling axis targeted by AD-1 [21]. Furthermore, in patients with RA, there is often abnormal activation of the PI3K signaling pathway. PI3K is one of the key factors in the anti-RA treatment [51]. In our previous work, it was found that AD-1 has a significant role in alleviating inflammation in IBD. Similarly, can AD-1 also have an alleviating effect on the inflammatory disease RA?

Network pharmacology is a theoretical concept that can effectively and clearly reveal the targets of drugs and diseases [52]. Molecular docking and simulation studies provide critical insights into the interaction mechanisms and conformational stability of protein–drug complexes [53]. Therefore, this study aimed to investigate the potential effects of AD-1 on RA and to validate the predicted molecular mechanisms from network pharmacology through in vitro experiments.

Firstly, overlapping targets were identified by intersecting 271 predicted AD-1 targets with 942 RA-related genes. Secondly, a PPI network was constructed, which identified 10 hub genes (SRC, MMP9, ELANE, SERPINA1, MAPK8, AKT1, PIK3R1, ALB, KDR and GSTP1). These genes are closely associated with the regulation of signal transduction, protein phosphorylation, cell proliferation, migration, and apoptosis. KEGG pathway enrichment analysis highlighted the PI3K/Akt signaling pathway as one of the most prominent pathways involved [54]. Moreover, GO enrichment analysis further identified PIK3R1 and AKT1 as the genes with the highest edge count. The molecular docking results revealed that AD-1 exhibited strong binding affinity toward PIK3R1 and AKT1. Subsequent molecular dynamics simulation results demonstrated that the complexes formed after AD-1 binding showed similar trends in terms of conformational stability, protein tightness and wave behavior. The results of time-dependent RMSD, RoG, SASA, H-bond number, and RMSF analysis indicated that both systems achieve equilibrium. These findings indicate that AD-1 may exert its modulatory effects on RA cell behavior via direct binding to essential mediators within the PI3K/Akt signaling pathway.

Based on the previous research, a series of experiments were conducted to verify our findings. Firstly, existing studies have shown that the proliferation of RA-FLS and the increased secretion of inflammatory cytokines directly contribute to joint destruction [55,56]. In this study, through cell viability measurement, it was found that AD-1 has an anti-proliferative effect on MH7A cells. Secondly, RA progression critically involves the migration of RA-FLS toward cartilage and their infiltration into the surrounding extracellular matrix [57]. Furthermore, the secretion of inflammatory factors by FLS has been shown to promote a tumor-like phenotype, leading to cartilage invasion and degeneration, and ultimately bone injury [58]. The results of this study demonstrated that AD-1 effectively inhibited the migration and invasion of MH7A cells.

RA-FLS possess potent anti-apoptotic capacity, which contributes to the aggressive growth of pannus tissue [59]. Within the mitochondrial apoptosis pathway, the Bcl-2 protein family is a central regulator, where Bax acts as a pro-apoptotic member that modulates mitochondrial membrane permeability and facilitates the activation of downstream apoptotic factors [60]. Additionally, PI3K-activated Akt has been demonstrated to phosphorylate downstream targets such as Bax and Bcl-2, thereby modulating their anti-apoptotic functions and ultimately affecting apoptosis [5]. In this study, we sought to determine whether AD-1 could induce apoptosis in MH7A cells. The findings revealed that AD-1 markedly downregulated Bcl-2 expression while upregulating Bax levels, indicating its ability to induce apoptosis in MH7A cells. Collectively, these results suggest that AD-1 exerts anti-proliferative, anti-migratory, anti-invasion, and pro-apoptotic effects on MH7A cells. However, the precise molecular mechanisms underlying these effects require further elucidation.

In recent years, the PI3K/Akt signaling pathway has been recognized as a pivotal regulator in various cellular processes and has emerged as a prominent pharmacological target in the treatment of RA, representing one of the most active areas of inflammation research [51]. PI3K, composed of a catalytic subunit (p110) and a regulatory subunit (p85), is activated by upstream cell surface receptors, leading to the generation of phosphatidylinositol-3,4,5-triphosphate (PIP3). PIP3 subsequently activates downstream effectors such as Akt, thereby regulating cell proliferation, migration, apoptosis, and signal transduction [61,62]. Moreover, aberrant activation of the PI3K/Akt pathway has been implicated in the pathogenesis and progression of various inflammatory and metabolic disorders, including RA, neuroinflammation, and meta-inflammation [63,64,65]. In this study, network pharmacology analysis predicted that AD-1 targets multiple genes within the PI3K/Akt pathway, with 10 overlapping genes identified, among which PIK3R1 and AKT1 were central nodes. This suggests a key role for this pathway in mediating the anti-RA effects of AD-1. Numerous studies have shown that the PI3K/Akt pathway is over-activated in RA and promotes disease progression [29,66,67]. To validate the results of the network pharmacology, we searched the RABC database and found that PIK3R1 and AKT1 were highly expressed in RA. Our Western blot analysis demonstrated that AD-1 significantly reduced the phosphorylation levels of PI3K and Akt, without markedly affecting the total protein levels of PI3K or Akt. This suggests that AD-1 suppresses the activation of the PI3K/Akt signaling cascade rather than altering gene expression. In addition, pharmacological modulation using the PI3K inhibitor LY294002 and the activator 740Y-P validated that PI3K/Akt signaling is directly involved in AD-1’s mechanism of action. As Akt functions downstream of PI3K, the reduction in p-Akt observed after AD-1 treatment is most likely secondary to upstream PI3K inhibition rather than a change in total Akt protein. This interpretation is supported by decreased p-PI3K/PI3K and p-Akt/Akt ratios, which reflect pathway activation status rather than protein expression levels [68,69,70]. AKT is downstream of PI3K (P13K/PKD/AKT) in the signaling pathway. The inhibition of AKT phosphorylation is likely secondary to P13K inhibition by AD-1. Collectively, these results indicate that inhibition of the PI3K/Akt pathway constitutes a critical component of the anti-inflammatory and anti-proliferative effects of AD-1 in RA. The results of TUNEL immunofluorescence and WB showed that AD-1 induced apoptosis of MH7A cells by up-regulating the pro-apoptotic protein Bax and down-regulating the anti-apoptotic protein Bcl-2. In addition, the wound healing experiments and the Transwell experiments demonstrated that AD-1 could effectively inhibit the migration and invasion abilities of MH7A cells. Finally, pathway activation in this study was inferred from phospho-to-total ratios of PI3K and Akt together with pharmacological modulation using LY294002 and 740Y-P, which are accepted readouts of signaling status but do not directly quantify catalytic activity or target engagement; accordingly, we did not directly measure PI3K or AKT enzyme activity nor assess target engagement. Future work will include PI3K lipid-kinase assays to quantify PIP3 production, immunoprecipitation-based AKT kinase assays with GSK-3 or Crosstide substrates, orthogonal readouts such as PIP3 ELISA or lipidomics, target-engagement methods such as the cellular thermal shift assay or drug-affinity responsive target stability, and genetic or pharmacological rescue experiments using myristoylated AKT or SC79 and isoform-selective PI3K inhibitors to further validate the mechanism.

## 5. Conclusions

In summary, this study used network pharmacology, molecular docking and molecular dynamics methods, combined with in vitro experiments for verification, to find that AD-1 inhibits the proliferation, migration and invasion of MH7A cells, while promoting apoptosis, by regulating the PI3K/Akt signaling pathway. These results provide an experimental basis for the further development of ginsenoside AD-1 as a potential drug for the treatment of RA.

## Figures and Tables

**Table 1:** Potential target genes of AD-1 for RA.

Gene Symbol								
ALB	GSTP1	PIK3CG	HSPA8	REN	CYP2C9	MTHFD1	PIK3R1	LGALS3
MAPK1	ADAM17	ESR1	PDE4D	FGF1	JAK3	VDR	TEK	TGFB2
BMP2	MAPK8	SRC	DPP4	ELANE	NR3C1	ZAP70	S100A9	CTSB
AKR1B1	MMP13	CDK2	GSR	HMGCR	MMP2	DHFR	CTSG	MMP9
TTR	AR	KDR	PLA2G10	PGF	CTSK	PRKCQ	JAK2	GSTM1
GC	PPARG	TYMS	SYK	PLA2G2A	PPARA	HSP90AB1	XIAP	HGF
CYP19A1	MMP3	DHODH	FGFR1	PARP1	IL2	ACE	CBS	BCL2L1
ITGAL	EGFR	NOS3	RBP4	CDK6	SERPINA1	CAT	KIT	BTK
F2	MAPK14	ANXA5	ESRRA	MMP12	IGF1	BPI	HMOX1	AKT1
CASP3	WAS	LCK	MMP8	SOD2	PADI4	PPP1CC	CASP1	

**Table 2:** Top 10 targets of AD-1 against RA.

Number	Gene Symbol	Uniprot ID	Gene Full Name
1	SRC	P12931	Src Proto-Oncogene, Non-Receptor Tyrosine Kinase
2	MMP9	P14780	Matrix Metallopeptidase 9
3	ELANE	P08246	Elastase, Neutrophil Expressed
4	SERPINA1	P01009	Serpin Family A Member 1
5	MAPK8	P45983	Mitogen-Activated Protein Kinase 8
6	AKT1	P31749	AKT Serine/Threonine Kinase 1
7	PIK3R1	P27986	Phosphoinositide-3-Kinase Regulatory Subunit 1
8	ALB	P02768	Albumin
9	KDR	P35986	Kinase Insert Domain Receptor
10	GSTP1	P09211	Glutathione S-Transferase Pi 1

**Table 3:** Result of molecular docking.

Targets	PDB	Binding Energy (kcal/mol)
PIK3R1	4WAF	−8.1 kcal/mol
AKT1	3QKM	−8.4 kcal/mol

**Table 4:** The MM/GBSA results of AD-1.

Targets (PDB)	PIK3R1 (4WAF)			AKT1 (3QKM)		
	HOLO-1	HOLO-2	HOLO-3	HOLO-1	HOLO-2	HOLO-3
**E_VDW_**	−51.97 ± 2.01	−51.35 ± 4.66	−53.03 ± 2.23	−50.25 ± 3.67	−41.35 ± 6.26	−45.30 ± 4.10
**E_ELE_**	−26.40 ± 9.35	−25.03 ± 4.40	−18.57 ± 5.79	−16.69 ± 8.46	−18.37 ± 6.83	−17.50 ± 7.20
**E_GB_**	46.48 ± 5.22	46.77 ± 5.90	44.99 ± 3.55	38.46 ± 8.18	36.14 ± 5.54	37.30 ± 6.00
**E_SA_**	−6.72 ± 0.34	−6.79 ± 0.60	−6.26 ± 0.46	−6.73 ± 0.60	−5.05 ± 0.87	−5.90 ± 0.75
**Bind energy**	−38.60 ± 5.56	−36.39 ± 3.14	−32.87 ± 4.43	−35.22 ± 3.20	−28.63 ± 7.41	−31.40 ± 4.80

Note: E_VDW_: van der Waals energy; E_ELE_: electrostatic energy; E_GB_: electrostatic contribution to solvation; E_SA_: non-polar contribution to solvation.

## Data Availability

Data will be made available on request.

## Supplementary Materials

The following supporting information can be downloaded at https://www.mdpi.com/article/10.3390/cells14201625/s1. Table S1: Result of molecular docking.

## References

[1] Di Matteo A., Bathon J.M., Emery P. (2023). Rheumatoid arthritis. Lancet.

[2] Bergstra S.A., Sepriano A., Kerschbaumer A., van der Heijde D., Caporali R., Edwards C.J., Verschueren P., de Souza S., Pope J.E., Takeuchi T. (2023). Efficacy, duration of use and safety of glucocorticoids: A systematic literature review informing the 2022 update of the EULAR recommendations for the management of rheumatoid arthritis. Ann. Rheum. Dis..

[3] Kondo N., Kuroda T., Kobayashi D. (2021). Cytokine networks in the pathogenesis of rheumatoid arthritis. Int. J. Mol. Sci..

[4] Smolen J.S., Landewé R.B.M., Bergstra S.A., Kerschbaumer A., Sepriano A., Aletaha D., Caporali R., Edwards C.J., Hyrich K.L., Pope J.E. (2023). EULAR recommendations for the management of rheumatoid arthritis with synthetic and biological disease-modifying antirheumatic drugs: 2022 update. Ann. Rheum. Dis..

[5] Liu S., Ma H.X., Zhang H.X., Deng C.J., Xin P. (2021). Recent advances on signaling pathways and their inhibitors in rheumatoid arthritis. Clin. Immunol..

[6] Galvano F., La Fauci L., Lazzarino G., Fogliano V., Ritieni A., Ciappellano S., Battistini N.C., Tavazzi B., Galvano G. (2004). Cyanidins: Metabolism and biological properties. J. Nutr. Biochem..

[7] Samarpita S., Ganesan R., Rasool M. (2020). Cyanidin prevents the hyperproliferative potential of fibroblast-like synoviocytes and disease progression via targeting IL-17A cytokine signalling in rheumatoid arthritis. Toxicol. Appl. Pharmacol..

[8] Yang J.Y., Yang H., Wang F.M., Dai Y., Deng Y.X., Shi K.Y., Zhu Z.H., Liu X.K., Ma X., Gao Y.X. (2025). Bioinformatics identification based on causal association inference using multi-omics reveals the underlying mechanism of Gui-Zhi-Shao-Yao-Zhi-Mu decoction in modulating rheumatoid arthritis. Phytomedicine.

[9] Xin P., Xu X.Y., Zhang H.X., Hu Y.Z., Deng C.J., Sun S.Q., Liu S., Zhou X.G., Ma H.X., Li X.L. (2023). Mechanism investigation of Duhuo Jisheng pill against rheumatoid arthritis based on a strategy for the integration of network pharmacology, molecular docking and in vivo experimental verification. Pharm. Biol..

[10] Bao J.M., Song Y.J., Hang M.H., Xu H., Li Q., Wang P.Y., Chen T., Xia M.X., Shi Q., Wang Y.J. (2024). Huangqi Guizhi Wuwu Decoction suppresses inflammation and bone destruction in collagen-induced arthritis mice. Chin. Herb. Med..

[11] Radu A., Bungau S.G. (2021). Management of rheumatoid arthritis: An overview. Cells.

[12] Ren B., Feng J., Yang N., Guo Y., Chen C., Qin Q. (2021). Ginsenoside Rg3 attenuates angiotensin II-induced myocardial hypertrophy through repressing NLRP3 inflammasome and oxidative stress via modulating SIRT1/NF-κB pathway. Int. Immunopharmacol..

[13] Wei Y.G., Yang H.X., Zhu C.H., Deng J.J., Fan D.D. (2020). Hypoglycemic effect of ginsenoside Rg5 mediated partly by modulating gut microbiota dysbiosis in diabetic db/db mice. J. Agric. Food Chem..

[14] Tang M.S., Xie X., Yang Y.Y., Li F. (2021). Ginsenoside compound K- a potential drug for rheumatoid arthritis. Pharmacol. Res..

[15] Zhang Y., Wang S., Song S., Yang X.M., Jin G. (2020). Ginsenoside Rg3 alleviates complete Freund's adjuvant-induced rheumatoid arthritis in mice by regulating CD4^+^ CD25^+^ Foxp3^+^ Treg cells. J. Agric. Food Chem..

[16] Wang W., Zhao Y., Rayburn E.R., Hill D.L., Wang H., Zhang R. (2007). In vitro anti-cancer activity and structure-activity relationships of natural products isolated from fruits of *Panax ginseng*. Cancer Chemother. Pharmacol..

[17] Zhao Y., Wang W., Han L., Rayburn E.R., Hill D.L., Wang H., Zhang R. (2007). Isolation, structural determination, and evaluation of the biological activity of 20(S)-25-methoxyl-dammarane-3beta, 12beta, 20-triol [20(S)-25-OCH_3_-PPD], a novel natural product from *Panax notoginseng*. Med. Chem..

[18] Zhao J.M., Li N., Zhang H., Wu C.F., Piao H.R., Zhao Y.Q. (2011). Novel dammarane-type sapogenins from *Panax ginseng* berry and their biological activities. Bioorg. Med. Chem. Lett..

[19] Ding M., Lu J., Zhao C., Zhang S., Zhao Y. (2016). Determination of 25-OCH_3_-PPD and the related substances by UPLC-MS/MS and their cytotoxic activity. J. Chromatogr. B..

[20] Li T., Chen Y., Li Y., Chen G., Zhao Y.Q., Su G. (2022). Antifibrotic effect of AD-1 on lipopolysaccharide-mediated fibroblast injury in L929 cells and bleomycin-induced pulmonary fibrosis in mice. Food Funct..

[21] Li J.W., Li F.F., Zhao Y.Q., Jin D. (2023). Integrating network pharmacology and experimental validation to explore the effect and mechanism of AD-1 in the treatment of colorectal cancer. Front. Pharmacol..

[22] Zhang L.H., Jia Y.L., Lin X.X., Zhang H.Q., Dong X.W., Zhao J.M., Shen J., Shen H.J., Li F.F., Yan X.F. (2013). AD-1, a novel ginsenoside derivative, shows anti-lung cancer activity via activation of p38 MAPK pathway and generation of reactive oxygen species. Biochim. Biophys. Acta.

[23] Wang X., Shen Y.H., Wang S.W., Li S.L., Zhang W.L., Liu X.F., Lai L.H., Pei J.F., Li H.L. (2017). PharmMapper 2017 update: A web server for potential drug target identification with a comprehensive target pharmacophore database. Nucleic. Acids Res..

[24] Alharbi K.S., Shaikh M.A.J., Almalki W.H., Kazmi I., Al-Abbasi F.A., Alzarea S.I., Imam S.S., Alshehri S., Ghoneim M.M., Singh S.K. (2022). PI3K/Akt/mTOR pathways inhibitors with potential prospects in non-small-cell lung cancer. J. Environ. Pathol. Tox..

[25] Rebhan M., Chalifa-Caspi V., Prilusky J., Lancet D. (1997). GeneCards: Integrating information about genes, proteins and diseases. Trends Genet..

[26] Szklarczyk D., Kirsch R., Koutrouli M., Nastou K., Mehryary F., Hachilif R., Gable A.L., Fang T., Doncheva N.T., Pyysalo S. (2023). The STRING database in 2023: Protein–protein association networks and functional enrichment analyses for any sequenced genome of interest. Nucleic. Acids Res..

[27] Sherman B.T., Hao M., Qiu J., Jiao X.L., Baseler M.W., Lane H.C., Imamichi T., Chang W.Z. (2022). DAVID: A web server for functional enrichment analysis and functional annotation of gene lists (2021 update). Nucleic. Acids Res..

[28] Bu D.C., Luo H.T., Huo P.P., Wang Z.H., Zhang S., He Z.H., Wu Y., Zhao L.H., Liu J.J., Guo J.C. (2021). KOBAS-i: Intelligent prioritization and exploratory visualization of biological functions for gene enrichment analysis. Nucleic. Acids Res..

[29] Jiang C.H., Zeng X., Wang J., Wu X.Q., Song L.J., Yang L., Li Z., Xie N., Yuan X.M., Wei Z.F. (2025). Andrographolide sulfonate alleviates rheumatoid arthritis by inhibiting glycolysis-mediated activation of PI3K/AKT to restrain Th17 cell differentiation. Chin. J. Nat. Med..

[30] Sagui C., Darden T.A. (1999). Molecular dynamics simulations of biomolecules: Long-range electrostatic effects. Annu. Rev. Biophys. Biomol. Struct..

[31] Kr Utler V., van Gunsteren W.F., Hünenberger P.H. (2001). A fast SHAKE algorithm to solve distance constraint equations for small molecules in molecular dynamics simulations. J. Comput. Chem..

[32] Zhang S., Tang Y., Cao J., Zhao C., Zhao Y. (2015). Crystallization-induced dynamic resolution R-epimer from 25-OCH_3_-PPD epimeric mixture. J. Chromatogr. B.

[33] Ding M., Wang X., Zhang Y., Yuan W., Zhang H., Xu L., Wang Z., Lu J., Li W., Zhao Y. (2019). New perspective on the metabolism of AD-1 in vivo: Characterization of a series of dammarane-type derivatives with novel metabolic sites and anticancer mechanisms of active oleanane-type metabolites. Bioorganic Chem..

[34] Fang X.Z., Lee Y.H., Jang J.H., Kim S.J., Kim S.H., Kim D.H., Na H.K., Kim K.O., Baek J.H., Surh Y.J. (2023). ARD1 stabilizes NRF2 through direct interaction and promotes colon cancer progression. Life Sci..

[35] Kitchen D.B., Decornez H., Furr J.R., Bajorath J. (2004). Docking and scoring in virtual screening for drug discovery: Methods and applications. Nat. Rev. Drug Discov..

[36] Chang M.W., Lindstrom W., Olson A.J., Belew R.K. (2007). Analysis of HIV wild-type and mutant structures via in silico docking against diverse ligand libraries. J. Chem. Inf. Model..

[37] Swargiary A., Roy M.K., Mahmud S. (2023). Phenolic compounds as alpha-glucosidase inhibitors: A docking and molecular dynamics simulation study. J. Biomol. Struct. Dyn..

[38] Sadeghi M., Miroliaei M., Ghanadian M., Szumny A., Rahimmalek M. (2023). Exploring the inhibitory properties of biflavonoids on alpha-glucosidase; computational and experimental approaches. Int. J. Biol. Macromol..

[39] Yang T., Yang Z., Pan F., Jia Y., Cai S., Zhao L., Zhao L., Wang O., Wang C. (2022). Construction of an MLR-QSAR Model Based on Dietary Flavonoids and Screening of Natural alpha-Glucosidase Inhibitors. Foods.

[40] Yu Z., Kan R., Ji H., Wu S., Zhao W., Shuian D., Liu J., Li J. (2021). Identification of tuna protein-derived peptides as potent SARS-CoV-2 inhibitors via molecular docking and molecular dynamic simulation. Food Chem..

[41] Pan J., Nawaz M., Liu J., Liu H., Lv Z., Yang W., Jiao Z., Zhang Q. (2025). Exploring synergistic inhibitory mechanisms of flavonoid mixtures on alpha-glucosidase by experimental analysis and molecular dynamics simulation. Food Chem..

[42] Brown P., Pratt A.G., Hyrich K.L. (2024). Therapeutic advances in rheumatoid arthritis. BMJ Br. Med. J..

[43] Wang X.W., Ni T.Y., Miao J.R., Huang X.Y., Feng Z. (2025). The role and mechanism of triptolide, a potential new DMARD, in the treatment of rheumatoid arthritis. Ageing Res. Rev..

[44] Pan D.M., Guo Y., Liu Y., Yang H.X., Gong Z.H., Du Y.Y., Xu R.T., Gao L., Xu Q., Li N. (2025). Guizhi Shaoyao Zhimu decoction alleviates rheumatoid arthritis by inhibiting inflammation by targeting SLPI. Phytomedicine.

[45] Ren M., Ma K., Pang X., Liu Y., Song Z., Zhou R., Tang Z. (2023). Anti-rheumatoid arthritis effects of total saponins from Rhizoma Panacis Majoris on adjuvant-induced arthritis in rats and rheumatoid arthritis fibroblast-like synoviocytes. Phytomedicine.

[46] Jain S., Tripathi S., Tripathi P.K. (2023). Antioxidant and antiarthritic potential of berberine: In vitro and in vivo studies. Chin. Herb. Med..

[47] Wang Y., Chen J., Luo X., Zhang Y., Si M., Wu H., Yan C., Wei W. (2016). Ginsenoside metabolite compound K exerts joint-protective effect by interfering with synoviocyte function mediated by TNF-alpha and Tumor necrosis factor receptor type 2. Eur. J. Pharmacol..

[48] Yi Y.S. (2019). Ameliorative effects of ginseng and ginsenosides on rheumatic diseases. J. Ginseng Res..

[49] Ding M., Zhang Y., Wang X., Zhao Y. (2018). Gender-related differences in pharmacokinetics, tissue distribution, and excretion of 20(R)-25-methoxyl-dammarane-3beta,12beta,20-triol and its metabolite in rats and anti-ovarian cancer evaluation. J. Pharm. Biomed. Anal..

[50] Zhao C., Su G.Y., Wang X.D., Zhang X.S., Guo S., Zhao Y.Q. (2016). Antitumor activity of ginseng sapogenins, 25-OH-PPD and 25-OCH_3_-PPD, on gastric cancer cells. Biotechnol. Lett..

[51] Cheung T.T., McInnes I.B. (2017). Future therapeutic targets in rheumatoid arthritis?. Semin. Immunopathol..

[52] Nogales C., Mamdouh Z.M., List M., Kiel C., Casas A.I., Schmidt H.H.H.W. (2022). Network pharmacology: Curing causal mechanisms instead of treating symptoms. Trends Pharmacol. Sci..

[53] Basu A., Sarkar A., Maulik U. (2020). Molecular docking study of potential phytochemicals and their effects on the complex of SARS-CoV2 spike protein and human ACE2. Sci. Rep..

[54] Rommel C., Camps M., Ji H. (2007). PI3Kδ and PI3Kγ: Partners in crime in inflammation in rheumatoid arthritis and beyond?. Nat. Rev. Immunol..

[55] Zhang Q., Peng W., Wei S.J., Wei D.N., Li R.L., Liu J., Peng L.Y., Yang S., Gao Y.X., Wu C.J. (2019). Guizhi-Shaoyao-Zhimu decoction possesses anti-arthritic effects on type II collagen-induced arthritis in rats via suppression of inflammatory reactions, inhibition of invasion & migration and induction of apoptosis in synovial fibroblasts. Biomed. Pharmacother..

[56] Hong R., Sur B.J., Yeom M., Lee B., Kim K.S., Rodriguez J.P., Lee S., Kang K.S., Huh C.K., Lee S.C. (2018). Anti-inflammatory and anti-arthritic effects of the ethanolic extract of *Aralia continentalis* Kitag. in IL-1β-stimulated human fibroblast-like synoviocytes and rodent models of polyarthritis and nociception. Phytomedicine.

[57] Bottini N., Firestein G.S. (2013). Duality of fibroblast-like synoviocytes in RA: Passive responders and imprinted aggressors. Nat. Rev. Rheumatol..

[58] Nygaard G., Firestein G.S. (2020). Restoring synovial homeostasis in rheumatoid arthritis by targeting fibroblast-like synoviocytes. Nat. Rev. Rheumatol..

[59] McHugh J. (2020). SUMOylation links metabolic and aggressive phenotype of RA FLS. Nat. Rev. Rheumatol..

[60] Hird A.W., Tron A.E. (2019). Recent advances in the development of Mcl-1 inhibitors for cancer therapy. Pharmacol. Ther..

[61] Okkenhaug K. (2013). Signaling by the phosphoinositide 3-kinase family in immune cells. Annu. Rev. Immunol..

[62] Ruicci K.M., Plantinga P., Pinto N., Khan M.I., Stecho W., Dhaliwal S.S., Yoo J., Fung K., MacNeil D., Mymryk J.S. (2019). Disruption of the RICTOR/mTORC2 complex enhances the response of head and neck squamous cell carcinoma cells to PI3K inhibition. Mol. Oncol..

[63] Li M.Y., Tian F., Guo J.L., Li X.K., Ma L., Jiang M.M., Zhao J. (2023). Therapeutic potential of *Coptis chinensis* for arthritis with underlying mechanisms. Front. Pharmacol..

[64] Wright B., King S., Suphioglu C. (2024). The Importance of phosphoinositide 3-kinase in neuroinflammation. Int. J. Mol. Sci..

[65] Acosta-Martinez M., Cabail M.Z. (2022). The PI3K/Akt pathway in meta-inflammation. Int. J. Mol. Sci..

[66] Zhang Y.M., Jin H.Z., Jia W.Y., Liu Y.Q., Wang Y.R., Xue S.Y., Liu Y., Hao H.Q. (2025). Ermiao San attenuating rheumatoid arthritis via PI3K/AKT/mTOR signaling activate HIF-1α induced glycolysis. J. Ethnopharmacol..

[67] Wang F.F., Liu J. (2025). The dual anti-inflammatory and anticoagulant effects of Jianpi Huashi Tongluo prescription on rheumatoid arthritis through inhibiting the activation of the PI3K/AKT signaling pathway. Front. Pharmacol..

[68] Ding Q., Hu W., Wang R., Yang Q.Y., Zhu M.L., Li M., Cai J.H., Rose P., Mao J.C., Zhu Y.Z. (2023). Signaling pathways in rheumatoid arthritis: Implications for targeted therapy. Signal Transduct. Target. Ther..

[69] Fruman D.A., Chiu H., Hopkins B.D., Bagrodia S., Cantley L.C., Abraham R.T. (2017). The PI3K Pathway in Human Disease. Cell.

[70] Manning B.D., Toker A. (2017). AKT/PKB Signaling: Navigating the Network. Cell.

