# Supplementary material for: Ginsenoside Derivative AD-1 Suppresses Pathogenic Phenotypes of Rheumatoid Arthritis Fibroblast-like Synoviocytes by Modulating the PI3K/Akt Signaling Pathway
Source: Cells. 2025 Oct 18;14(20):1625. doi: 10.3390/cells14201625 (PMC12564127; doi:10.3390/cells14201625)
Supplement: Supplementary file 1 [file cells-14-01625-s001.zip › cells-3858013-supplementary.pdf]

Table S1. Result of molecular docking.

Targets (PDB)	Binding energy (kcal/mol)	Dist from best mode	
		rmsd l.b.	rmsd u.b.
PIK3R1 (4WAF)-1	-8.1	0.000	0.000
PIK3R1 (4WAF)-2	-8.1	1.776	3.308
PIK3R1 (4WAF)-3	-7.9	1.840	2.925
AKT1 (3QKM)-1	-8.4	0.000	0.000
AKT1 (3QKM)-2	-8.0	1.801	3.150
AKT1 (3QKM)-3	-7.6	2.333	8.577
